# Tuning cluster size down to single atoms on Pt/γ-Al_2_O_3_ catalysts *via* surface organometallic chemistry

**DOI:** 10.1039/d5sc04893a

**Published:** 2025-10-23

**Authors:** Martin Cotoni, Mickaël Rivallan, Isabelle Clémençon, Virgile Rouchon, Anne-Lise Taleb, Julie Poulizac, Amandine Cabiac, Christophe Bouchy, Christophe Copéret, Céline Chizallet

**Affiliations:** a IFP Energies Nouvelles, Rond-point de L'échangeur de Solaize BP3 69360 Solaize France mickael.rivallan@ifpen.fr celine.chizallet@ifpen.fr; b Department of Chemistry and Applied Biosciences, ETH Zürich Zürich CH-8093 Switzerland ccoperet@inorg.chem.ethz.ch

## Abstract

Platinum catalysts supported on γ-Al_2_O_3_ are central to a variety of applications. The conditions controlling the formation of Pt single atoms and subnanometric clusters remain elusive. The present work, based on surface organometallic chemistry (SOMC), unravels their formation under oxidative and reductive atmospheres. Following the grafting of MeCpPtMe_3_ as a molecular precursor to generate highly dispersed sites on alumina, the evolution upon thermal treatment under oxidative or reductive conditions is monitored by *in situ* FTIR, with the ultimate goal to access catalysts with different single atom-to-cluster ratios, comparing SOMC with a conventional preparation method. Under oxidative conditions, all ligands are removed to form CO_2_ in a multi-step process, while under reductive conditions, ligands likely decompose through hydrogenolysis/hydrogenation reactions. HAADF-STEM characterization and CO adsorption experiments reveal the presence of several states of Pt, depending on the Pt surface density and the treatment applied. Under a reductive atmosphere, the size of platinum clusters remains relatively constant and lower than 0.8 nm, regardless of the Pt surface density (0.03–0.09–0.15 Pt nm^−2^). Under an oxidative atmosphere, the Pt surface density is a key factor that drives the size of platinum clusters and the relative amount of single atoms, both of which are significantly different from those of a reference conventional catalyst obtained by incipient wetness impregnation of Pt(NH_3_)_4_(NO_3_)_2_. Notably, the material at 0.03 Pt nm^−2^ exhibits mainly Pt single atoms after calcination, while increasing Pt density favors cluster formation.

## Abstract 2

Pt/γ-Al_2_O_3_ catalysts are prepared by surface organometallic chemistry and treatment under oxidative or reductive atmospheres. They exhibit a smaller particle size and/or a higher single atom/cluster ratio than a conventional catalyst.

## Graphical abstract

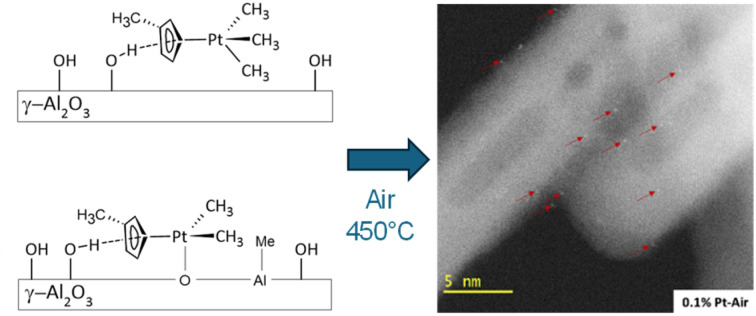

## Introduction

Pt/γ-Al_2_O_3_ systems are highly versatile catalysts commonly used in various fields such as abatement of automobile emissions,^1^ petrochemistry,^2,3^ oil refining,^4,5^ biomass conversion,^6–8^ dehydrogenation of liquid organic hydrogen carriers,^9,10^ and, more recently, in emerging applications such as catalytic recycling of plastics.^11,12^ A large set of methods of platinum deposition on the alumina support has been described, *e.g.* impregnation,^13,14^ deposition–precipitation,^15,16^ or adsorption^17,18^ of platinum salts. These methods rely on the use of aqueous solutions of inorganic precursors with complex deposition mechanisms. They often lead to particle sizes that approach 1 nm even at low loadings after reduction.^19^

In this context, the deposition of Pt using organometallic precursors represents an alternative approach for the controlled genesis of supported Pt catalysts,^20–23^ possibly to reach smaller particle sizes down to single atoms, paralleling the synthesis of single-site catalysts based on early transition metals.^24^ Pt organometallic precursors, such as trimethyl (methylcyclopentadiene)platinum(iv) (MeCpPtMe_3_), are used to prepare supported catalysts by atomic layer deposition (ALD)^25–29^ or chemical vapor deposition (CVD).^30,31^ These preparation techniques allow the controlled deposition of Pt on the support but require complex synthesis setups. A convenient alternative consists of using surface organometallic chemistry (SOMC), where molecular precursors are typically reacted at low temperature and in organic solvent selectively on highly reactive surface functionalities present at the support surface.^24,32^ Mainly described to prepare single-site catalysts, SOMC has also been used for the preparation of supported nanoparticles to interrogate the role of interfaces and compositions in heterogeneous catalysis.^33^ Contrary to gas phase deposition methods such as ALD, SOMC relies on a selective stoichiometric grafting reaction, avoiding the use of a large excess of precursor and high temperature, thereby minimizing secondary reactions. Furthermore, SOMC typically generates well-defined structures,^34^ suitable for detailed spectroscopic characterization, establishing structure–activity relationships as single-site catalysts or as starting materials to generate active phases in heterogeneous catalysis.^24^

The present work explores SOMC and key parameters to prepare highly dispersed Pt sites supported on γ-Al_2_O_3_ down to Pt single atoms, starting from MeCpPtMe_3_. This study focuses on how platinum density influences sintering under reductive or oxidative atmospheres, aiming to prepare Pt single-atom and subnanometric cluster catalysts supported on γ-Al_2_O_3_. *In situ* Fourier transformed infrared (FTIR), high-angle annular dark-field scanning transmission microscopy (HAADF-STEM) and CO adsorption measurements are conducted to understand the state of Pt, showing in particular that samples prepared by SOMC significantly differ from a reference sample prepared by a conventional technique (incipient wetness impregnation (IWI) using Pt(NH_3_)_4_(NO_3_)_2_ as a precursor), for which larger clusters are formed.^35–38^

## Results and discussion

### Characterization of the grafted materials

**Scheme 1:**
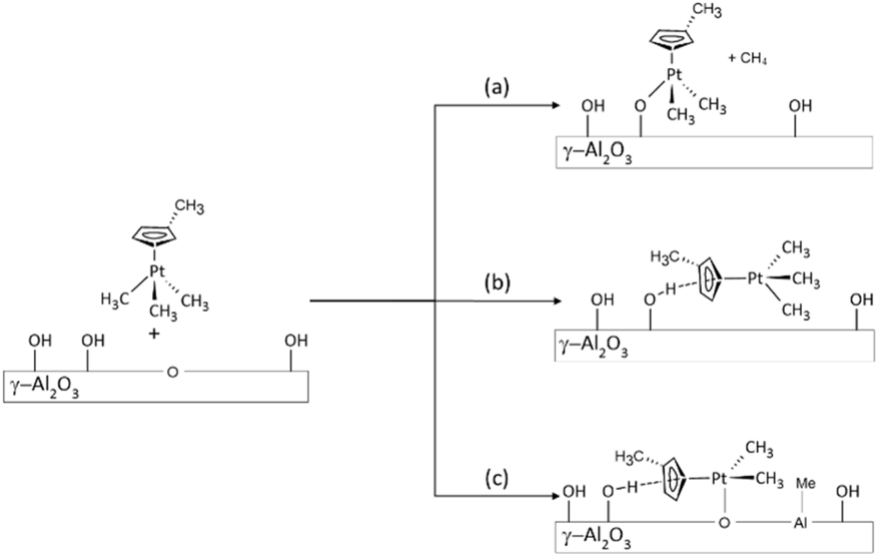
Possible interaction modes of the platinum precursor with the alumina surface: (a) protolysis and grafting, (b) hydrogen bonding interaction with the MeCp ligands, and (c) dissociation of a Pt–Me bond with Lewis acid/base sites of the surface.

A series of three catalysts is prepared by SOMC using Schlenk techniques under an argon atmosphere, with the following target Pt surface densities: 0.03–0.09–0.15 Pt nm^−2^, corresponding respectively to Pt loadings of 0.1–0.3–0.5 wt%. The alumina support prepared for this purpose exhibits a surface area of 104 m^2^ g^−1^ and a needle-like morphology (see the SI). Upon grafting, a protolysis reaction is typically expected between the reactive alumina surface hydroxyls and the M–C bond of the organometallic precursor (Scheme 1a),^39^ forming a M–O–Al bond and releasing methane. However, with MeCpPtMe_3_, no methane release is detected by ^1^H NMR while MeCpPtMe_3_ is strongly adsorbed (the grafting test procedure is given in the SI and NMR spectra are shown in Fig. S2).

**Fig. 1:**
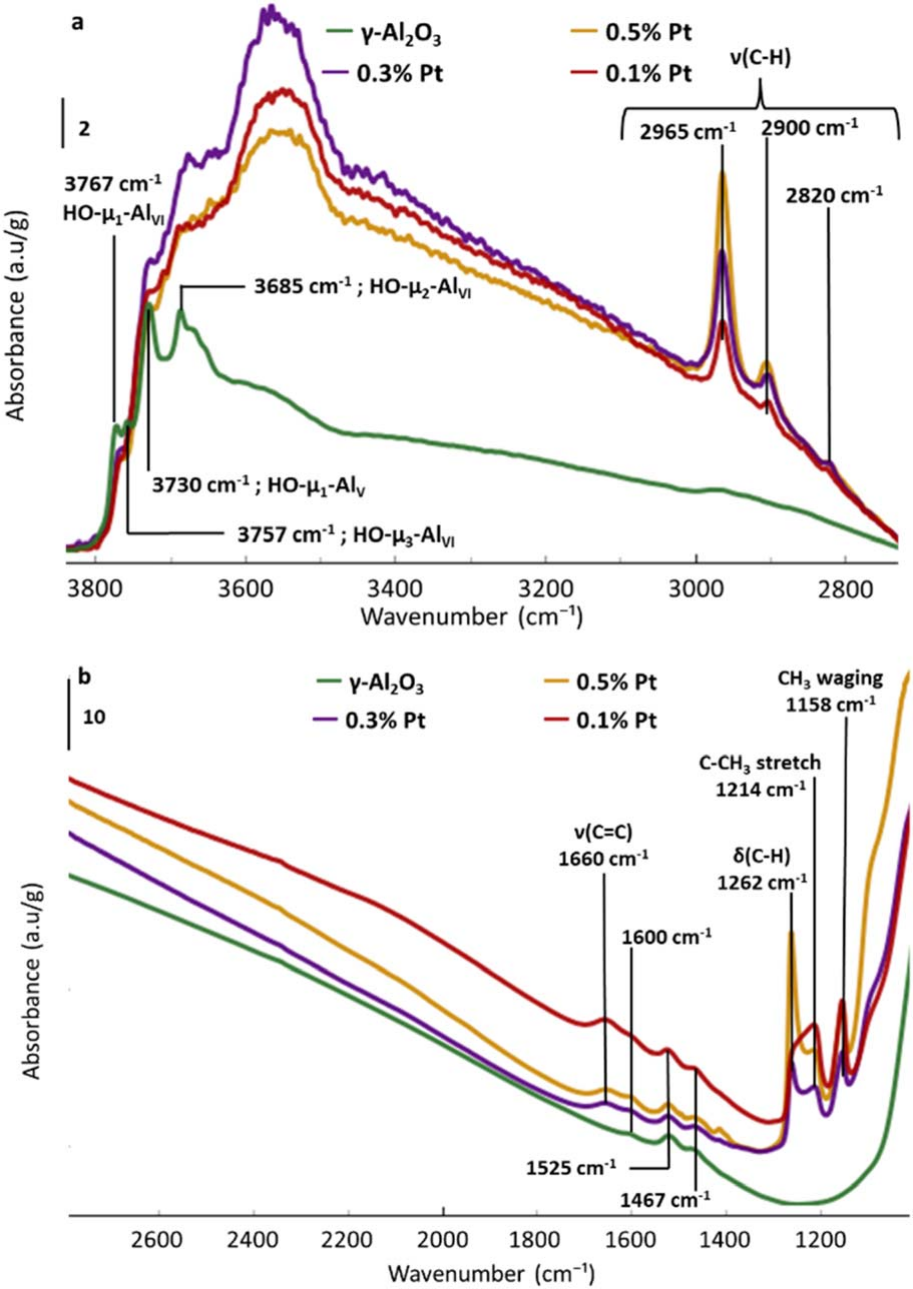
FTIR spectra of the MeCpPtMe_3_ precursor deposited at several Pt loadings (given in wt%) on γ-Al_2_O_3_ with a focus on (a) the OH and CH elongation zones (baselined corrected) and on (b) wavelength between 1000 and 2800 cm^−1^ (spectra are normalized by the sample mass).

FTIR is next employed to monitor the surface reactions (Fig. 1). The spectrum of the dehydrated alumina displays the typical OH vibration bands of γ-Al_2_O_3_ at 3767, 3757, 3730 and 3685 cm^−1^, assigned to various μ_*n*_–OH hydroxyl groups (*n* = 1–3) connected to Al_IV–V–VI_ surface aluminum atoms.^40–42^ Low intensity bands at 1467, 1525 and 1600 cm^−1^ are also present on the support (Fig. 1b). These bands are assigned to residual carbonate species and lauric acid derivatives, remaining from the aluminum precursor and the lauric acid used for the preparation of the alumina. Such signals have already been observed in the literature when organic compounds are used for the synthesis of the support.^43–46^ Notably, despite the absence of methane release, the organometallic precursor remains strongly adsorbed on alumina, even after several washing steps with pentane, as evidenced by *ν*(C–H),^47^ deformation and bending modes of the MeCp ligand present in MeCpPtMe_3_ (Fig. 1).^28,48^

Moreover, comparing the spectra of Pt impregnated alumina with the spectrum of the dehydrated alumina underlines the partial disappearance of hydroxyls at 3767 cm^−1^ associated with μ_1_ coordinated hydroxyls on Al_VI_ sites. This is accompanied by the appearance of a broad band (3500–3600 cm^−1^), indicative of the interaction between adsorbed MeCpPtMe_3_ and adjacent OH groups, likely explaining the loss/shift of the μ_1_–OH–Al_VI_ vibration at 3767 cm^−1^. The strong adsorption of MeCpPtMe_3_ evidenced by IR, along with the absence of methane formation during grafting, indicates that the adsorption of MeCpPtMe_3_ on alumina probably involves its interaction with hydroxyls through H-bonding and/or Lewis acid sites, as depicted in Scheme 1b and c.

### *In situ* FTIR monitored genesis of Pt catalysts

The materials are then thermally treated under dry air or H_2_. With the aim of better understanding the genesis of the Pt catalyst prepared by SOMC during post-treatment *via* calcination or reduction, *in situ* FTIR studies are also conducted on the 0.1% Pt sample. The reduction conditions chosen in the *in situ* infrared experiment (see the SI: atmospheric pressure, with a usual temperature ramp of 2 °C min^−1^ from room temperature to 450 °C with a step of 2 h at 450 °C) are representative of typical calcination/reduction experiments. Considering that the samples are exposed to air before the *in situ* FTIR study, spectra are recorded before and after air exposure (Fig. S3). Air exposure leads to the growth of a large signal in the OH vibration area and of another one centred at 1640 cm^−1^, attributed to physisorbed water. Very weak signals at 2081 cm^−1^ and 1366 cm^−1^ can be assigned to CO and formates, respectively, suggesting a (weak) partial oxidation of the precursor when exposed to air.^49,50^ No significant change is observed below 1300 cm^−1^. Therefore, except for rehydration, the exposure to air has a minor impact on the sample.

The signal between 1300 and 1200 cm^−1^ can be deconvolved (Fig. S4 and Table 1, which summarizes the assignments of all bands observed in the 1200–2000 cm^−1^ range) into three peaks at 1262, 1238 and 1214 cm^−1^. Signals at 1262 and 1214 cm^−1^, already observed in Fig. 1a, are assigned to CH_3_ deformation and C–CH_3_ stretching of MeCp, respectively, while the peak at 1238 cm^−1^ is also associated with C–CH_3_ stretching of MeCp.^28^

**Table 1:** Attribution of peaks observed by FTIR in the range 1000–2000 cm^−1^ on the 0.1% Pt sample

Entries	Peaks	Attribution	Ref.
1	1640 cm^−1^	Water	49 and 50
2	1158 cm^−1^	CH_3_ wagging of Pt–Me	28 and 48
3	1214 cm^−1^	C–CH_3_ stretching of MeCp	
	1238 cm^−1^		
4	1262 cm^−1^	CH_3_ deformation	
5	1600 cm^−1^	Carbonates and organic residues from the alumina synthesis	43 and 46
	1522 cm^−1^		
	1465 cm^−1^		

### Oxidative conditions

**Fig. 2:**
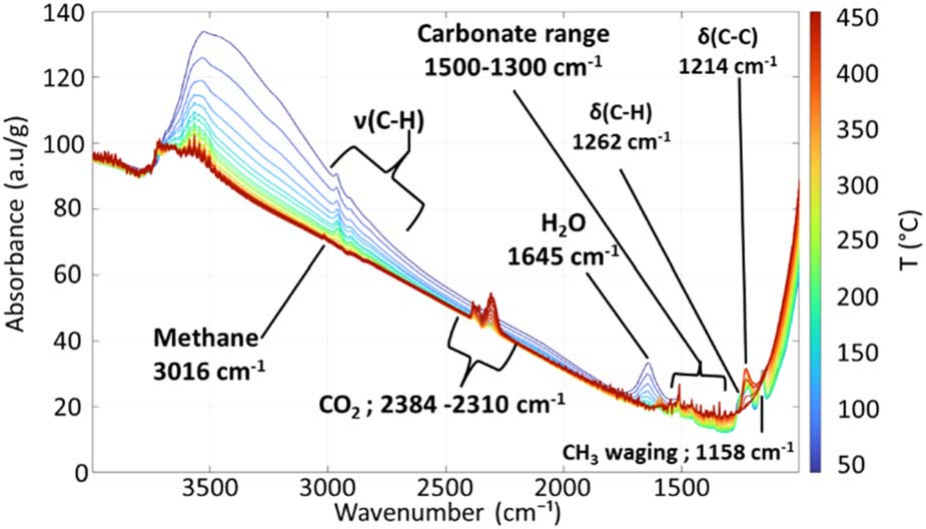
*In situ* FTIR spectra of the 0.1% Pt sample during calcination.

**Fig. 3:**
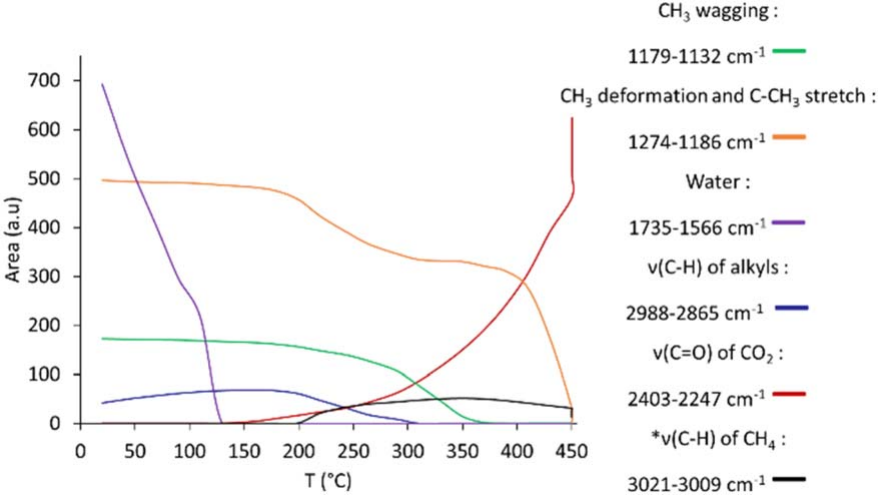
Evolution during calcination of the 0.1% Pt sample showing the area of observed signals (*corresponds to ten times the real area integrated for easier reading; integration interval given in the legend).

Fig. 2 and 3 present the evolution of FTIR spectra and the thermal evolution of the main signals observed during calcination monitored *in situ*, respectively. Overlapping signals at 1262 cm^−1^ and at 1214 cm^−1^ are integrated together. The carbonate/bicarbonate^49,51^ signal (1500–1300 cm^−1^) is not quantified because of its non-monotonous thermal evolution due to signals of the dehydrated alumina in the same area (entry 5 Table 1).

The detailed evolution of the spectra is discussed in the SI. The main observations can be rationalized as follows. CO_2_ is formed in the first place before methane, as already observed when preparing such materials by ALD.^52^ Between 170 °C and 200 °C, a partial combustion of ligands to form CO_2_ can first be invoked (eqn (1), 150–200 °C), that may compel the precursor to be more reactive towards the surface hydroxyl groups of the support, thereby forming methane above 200 °C by protolysis (eqn (2)), or through other mechanisms; *e.g.* methane formation has been observed during the decomposition of MeCpPtMe_3_ in the gas phase in the presence of oxygen.^28,47^1
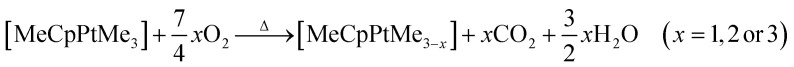
2
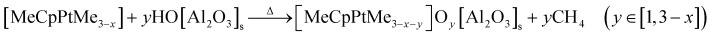


Above 350 °C, the decrease of the signal between 1274 and 1186 cm^−1^ indicates the decomposition of the MeCp ligand (being mainly attributed to C–CH_3_ deformation) into methane (eqn (3)) and CO_2_ (eqn (4)). During the plateau at 450 °C, the last remaining organic moieties are combusted into CO_2_, as indicated by the decrease of the methane signal and the increase of the CO_2_ signal.3
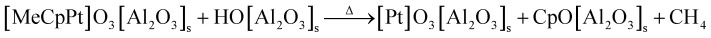
4
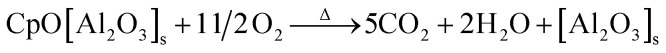


### Reductive conditions

**Fig. 4:**
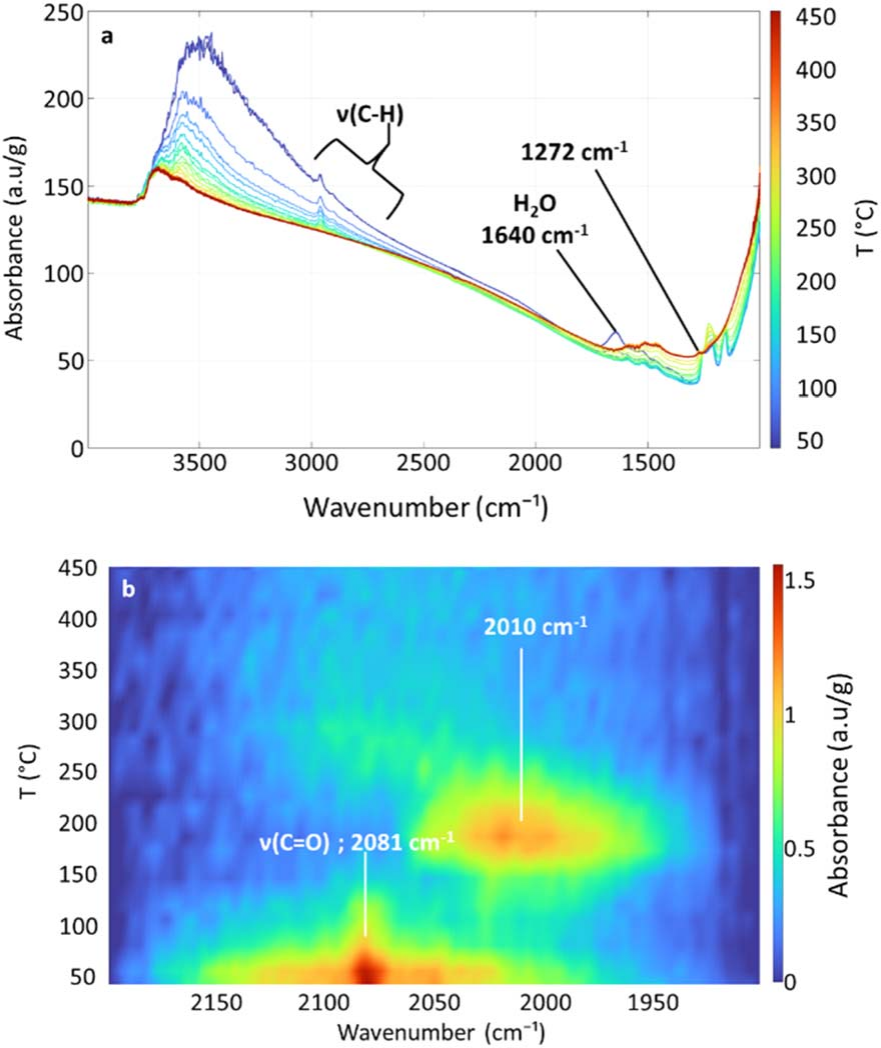
(a) *In situ* FTIR spectra of the 0.1% Pt sample during reduction and (b) mapping of the sample intensity as a function of temperature focused on the *ν*(C=O) area (2100–1900 cm^−1^, baseline corrected).

**Fig. 5:**
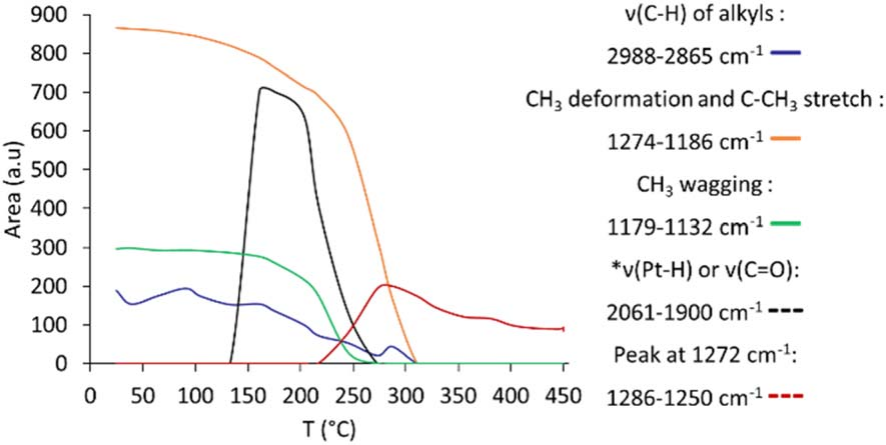
Evolution during reduction of the 0.1% Pt sample showing the area of observed signals (*corresponds to ten times the real area integrated for easier reading).

Fig. 4 shows the evolution of FTIR during the reduction, while Fig. 5 presents the integrated areas of observed signals during reduction as a function of temperature. A main difference with the treatment in an oxidative atmosphere is that no gaseous species (such as methane) are observed. Below 130 °C, pre-existing CO (Fig. 4b) and adsorbed water desorb. The *ν*(C–H) signal gradually decreases from room temperature to about 310 °C. The C–CH_3_ stretching and CH_3_ deformation signals slowly decrease up to 200 °C, where a much abrupt decrease starts. The slow decrease regime is accompanied by the appearance of a signal centred at 2010 cm^−1^ (Fig. 4b), starting from 130 °C, and reaching a maximum close to 160 °C. This signal can be assigned to adsorbed CO on platinum particles,^49,53^ or possibly to hydride species^54–56^ obtained by H_2_ dissociation on platinum particles. Regardless, both suggest that the reduction treatment generates clusters. The decrease of this signal above 160 °C suggests the desorption of the adsorbed CO/H_2_ from the clusters.

The CH_3_ wagging signal (1158 cm^−1^) abruptly decreases between 200 and 250 °C, while the C–CH_3_ stretching signal does the same between 250 and 310 °C. In these temperature intervals, a new weak signal at 1272 cm^−1^ appears (Fig. S5b). It can be attributed to alkoxy or aryl ether groups,^28,57^ possibly coming from surface reactions of methyl or Cp rings with surface hydroxyls or bridging oxygen groups. Notably, such species are not observed during calcination, possibly due to an important noisy baseline in this area, hence their formation as proposed in eqn 3 cannot be excluded. Under reduction conditions (Fig. 5), the concentration of these species reaches a maximum at 280 °C. They are not completely removed at the end of the thermal treatment.

Although the absence of CO_2_ under reduction conditions is expected, the absence of CH_4_ (or other alkanes that could be formed by hydrogenolysis and hydrogenation) is more surprising. It may be proposed that such species form but cannot be detected either in the gas phase (unlike under oxidative conditions) or as adsorbed species (like under oxidative conditions), but instead transform into surface alkoxy species as indicated by the signal at 1272 cm^−1^.

### Characterization of treated materials by HAADF-STEM

**Fig. 6:**
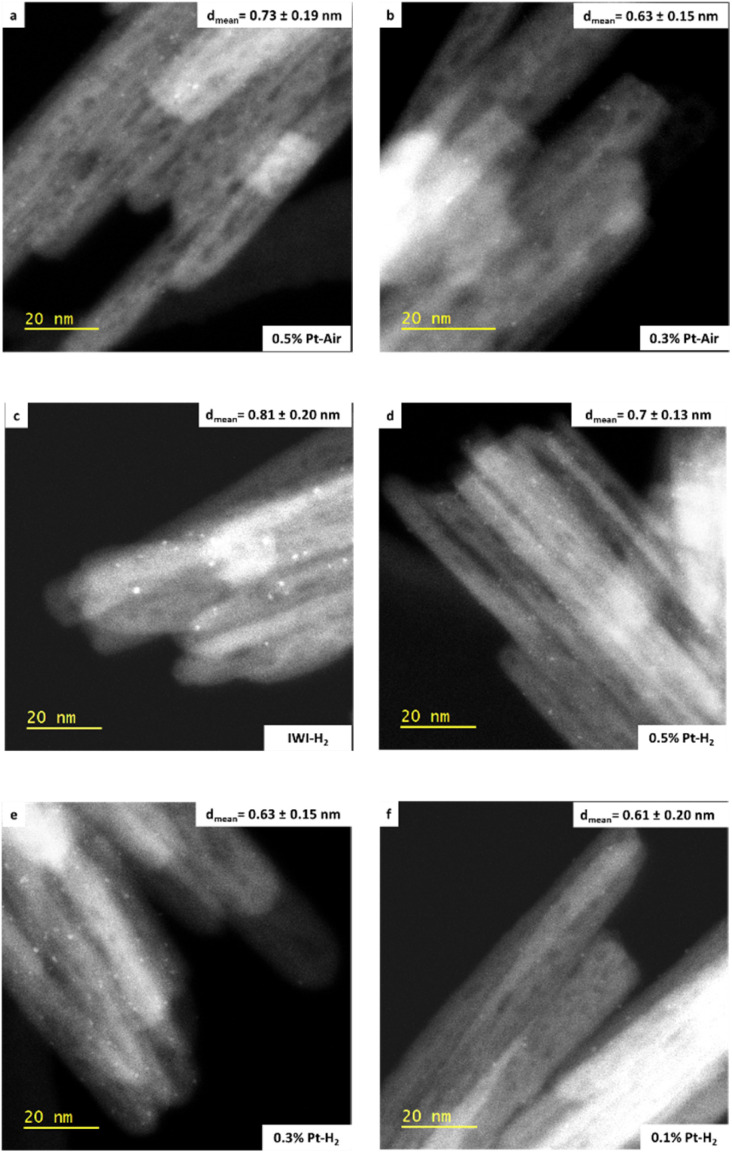
HAADF-STEM images obtained with a JEM-F200 microscope: (a) and (b) 0.3–0.5% Pt–Air, (c) IWI–H_2_ materials and (d)–(f) 0.5–0.3–0.1% Pt–H_2_.

Catalysts prepared by SOMC at various Pt loadings are then characterized using non-corrected HAADF-STEM to assess the aggregation state of Pt following each treatment at 450 °C (after 2 h plateau) (Fig. 6). The materials are denoted as *X*% Pt (*X* corresponding to Pt loading). “Air” or “H_2_” suffixes are added to indicate the thermal treatment applied. Moreover, their properties are compared to those of a conventional catalyst at 0.1% Pt prepared by IWI starting from the Pt(NH_3_)_4_(NO_3_)_2_ precursor, subject to the same thermal treatments (air or H_2_) as the samples prepared by SOMC.

**Fig. 7:**
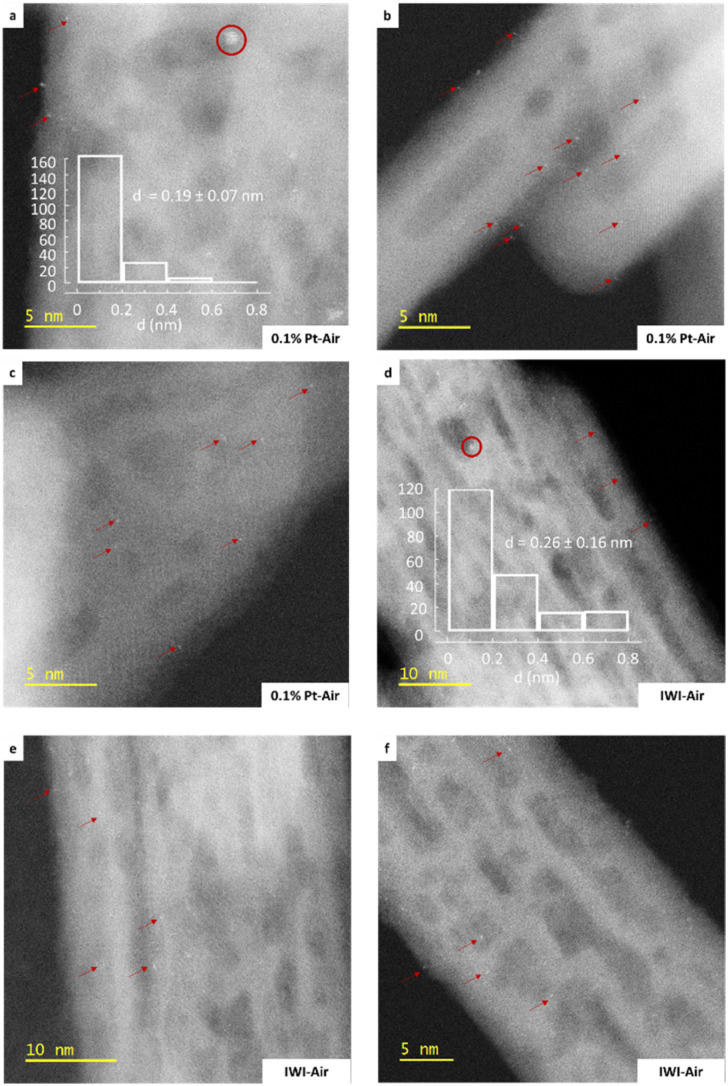
HR HAADF-STEM images obtained with a JEM-ARM200F microscope: 0.1% Pt–Air ((a)–(c)) and IWI–Air ((d)–(f)) samples (red arrows point single atoms and red circles indicate the presence of subnanometric clusters).

**Fig. 8:**
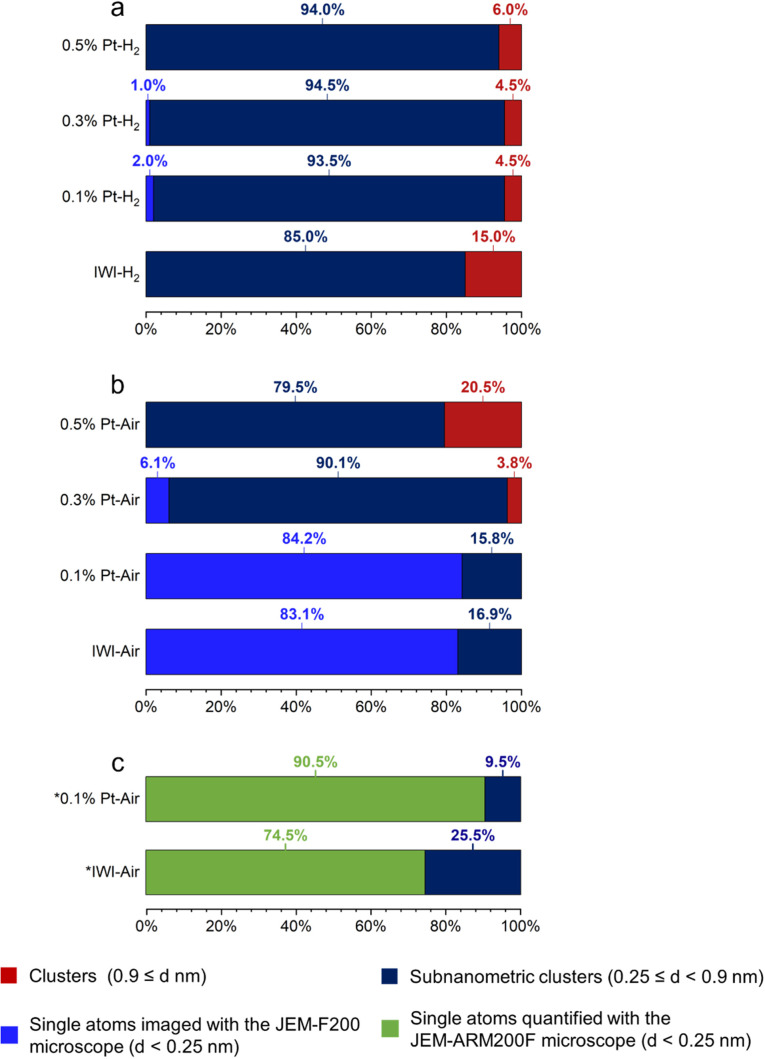
Size distribution of Pt objects characterized using a non-corrected microscope (JEM-F200) for reduced (a) and calcined (b) samples. Quantification is based on 200 objects, except for samples 0.1% Pt–Air and IWIAir, which are based on 76 and 60 objects, respectively. Since the JEM-F200 microscope is non-corrected, the quantification of the smallest objects (*d* < 0.25 nm) must be considered as inaccurate. (c) size distribution of Pt objects using an aberration-corrected microscope (JEM-ARM200F) for samples IWI–Air and 0.1% Pt–Air.

All samples dominantly exhibit clusters, except the 0.1% Pt–Air and IWI–Air samples, mainly composed of single atoms. Although the observation of single atoms is possible on non-corrected microscopes (Fig. S6),^58,59^ the latter two samples are also characterized using aberration-corrected HR HAADF-STEM to obtain better statistics on the distributions of the smallest objects (Fig. 7). Distributions of particle sizes (Fig. S7 and 7) are obtained for each material by measuring a total of 200 objects. For the SOMC calcined materials, the average particle size is larger as the Pt loading increases. This trend is also observed for the reduced materials, but to a much lesser extent. At 0.1% Pt loading, calcination promotes a smaller particle size than reduction. In contrast, the 0.3% Pt and 0.5% Pt exhibit similar particle size regardless of the treatment applied. The 0.1% Pt samples systematically exhibit a smaller particle size distribution compared to the IWI samples (0.26 and 0.81 nm for air and H_2_ treatment, respectively). To gain more detailed information on the different populations, measured objects are classified based on their sizes, with the following criteria: *d* < 0.25 nm, 0.25 ≤ *d* < 0.9 nm, 0.9 nm ≤ *d*, attributed to single atoms, subnanometric clusters and clusters, respectively. The data are reported in Fig. 8. The comparison between Fig. 8b and c substantiates the fact that the use of two microscopes with different resolutions induces a bias in the quantification.

This analysis reveals subtle differences between the 0.3% Pt and 0.5% Pt materials depending on the treatment. Indeed, air treatment tends to favour the formation of clusters for the higher platinum surface density (Fig. 8b). Conversely, a significant population of single atoms is observed in the 0.3% Pt–Air material (6.1% of the objects), while only a few of them (1% of the objects) are found on the reduced material at the same loading (Fig. 8a). Overall, the distribution of objects in the reduced samples prepared by SOMC is almost independent of the Pt loading, whereas a strong impact of the Pt loading is noted in the calcined samples.

Additionally, significant differences can be outlined from the comparison between the 0.1% Pt and IWI materials. After calcination, the most reliable analysis, made with a JEM-ARM200F microscope, shows that both materials exhibit a majority of Pt single atoms, but in a larger proportion for 0.1% Pt–Air (90.5%) than IWI–Air (74.5%) (Fig. 8c). It can also be noted that the proportion of clusters *vs.* subnanometric clusters differs between the 0.1% Pt–H_2_ and IWI–H_2_ samples, with a significant presence of clusters on the IWI–H_2_ sample. Eventually, the influence of the thermal treatment described earlier for the SOMC materials, also holds true for the materials prepared by IWI. However, the SOMC procedure presents the particularity to favour Pt dispersion compared to the IWI one, regardless of the treatment applied.

The preparation of the IWI material enables a clear comparison between a conventional preparation method and our proposed SOMC method for preparing single platinum atoms on alumina. Other techniques reported in the literature for preparing such materials can be further discussed. First, the work of Zhang *et al.*^60^ can be highlighted, where Pt_1_ atoms are stabilized by utilizing a mesoporous alumina support with a platinum loading of 0.2% (corresponding to a platinum density of 0.03 Pt nm^−2^). While this study emphasizes the importance of support engineering for the preparation of Pt_1_, our single-atom SOMC material offers the flexibility to be extended to other aluminas. However, it is worth noting that Zhang *et al.* successfully achieve Pt_1_ after reduction, whereas we do not. Atomic dispersion of Pt was achieved at a high platinum loading of 0.4% (corresponding to a platinum density of 0.06 Pt nm^−2^) by Wang *et al.*,^61^ thanks to the doping of the support with barium. The incorporation of a hetero-element into the support undoubtedly alters the resulting interactions between alumina and platinum. Our work, in contrast, provides a material that can be directly employed to investigate these interactions, thereby offering a more rational and precise understanding of the interaction between alumina and platinum.

The relationship between our findings and site stability under reaction conditions can be further assessed. Reactions such as CO oxidation are often performed at atmospheric total pressure and at temperatures far lower than 450 °C.^35^ Consequently, after a calcination treatment, it is expected that the single atoms and clusters formed during the pre-treatment will remain largely unaffected by the presence of O_2_ under test conditions, although the influence of CO pressure (not addressed in the present study) is likely significant. Some reactions led under H_2_ are performed under harsher conditions than the reduction protocol reported herein, while some others are not. For example, catalytic reforming is typically operated at temperatures above 500 °C and P(H_2_) ∼10 bar.^4^ In contrast, the dehydrogenation of methylcyclohexane (as a liquid organic hydrogen carrier) to toluene is typically performed at temperatures below 300 °C and atmospheric total pressure (hence P(H_2_) < 1 bar).^62^ Thus, the stability of the catalysts synthesized by our method will strongly depend on the operating conditions selected for the catalytic reaction in question. Stronger clusterization of single atoms and formation of nanoparticles from clusters are to be expected if severe reaction conditions are chosen. However, it is worth noting that this behaviour is also anticipated for samples obtained through conventional methods.

### Characterization of treated materials by CO adsorption monitored by IR

**Fig. 9:**
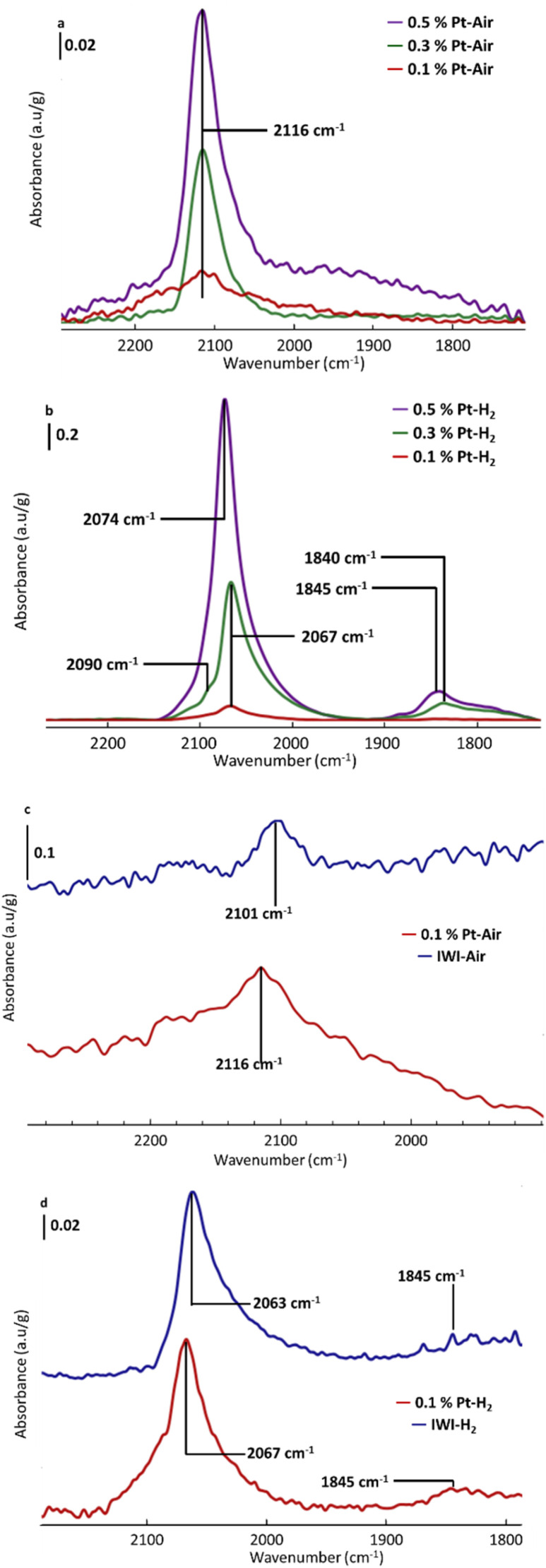
FTIR spectra after CO adsorption on: (a) Pt–Air samples, (b) Pt–H_2_ samples, (c) 0.1% Pt–Air compared to IWI–Air, and (d) 0.1% Pt– H_2_ compared to IWI–H_2_.

CO adsorption experiments are next conducted on all treated materials (Fig. 9). The calcined samples exhibit a weak signal at 2116 cm^−1^, whose intensity increases with platinum loading, except for IWI–Air with a red-shifted signal at 2101 cm^−1^.

According to previous experimental and density functional theory investigations,^49^ CO adsorbed on oxide Pt_13_ subnanometric clusters vibrates near 2100 cm^−1^. For CO adsorbed on oxide platinum single atoms, the signal is expected between 2094 and 2124 cm^−1^, depending on the specific environment of the single atom. Notably, a signal at 2119 cm^−1^ has been computed for CO linearly adsorbed on platinum on a Pt_1_O_2_ species. Thus, it is challenging to distinguish between signals from CO adsorbed on oxide clusters and those on oxide single atoms. This is consistent with the observation that, for all calcined SOMC materials, the signals are centred at 2116 cm^−1^.

Moreover, signals associated with partially oxidized clusters are linked to a shoulder around 2090–2070 cm^−1^ based on experimental data found in the literature.^35^ Considering the information provided by the STEM analysis, and the evolution of the IR intensity of the CO stretching band as a function of the platinum loading, only in the case of the 0.1% Pt–Air and IWI–Air samples this band can be attributed to platinum single atoms, with detectable differences in the environments of those single atoms from one catalyst to the other. For the two other calcined materials, the 2116 cm^−1^ signal most likely corresponds to CO linearly adsorbed on platinum oxide clusters.

Regarding the reduced materials, the spectra display peaks corresponding to CO linearly adsorbed on steps and low-coordinated sites of reduced platinum subnanometric clusters (∼2070 cm^−1^), on terrace (∼2090 cm^−1^) of Pt nanoparticles and multi-coordinated CO (∼1840 cm^−1^),^50–53^ with many more defined weak peaks for the IWI–H_2_ sample. Moreover, slight differences can be observed depending on the Pt density for the SOMC materials. Firstly, the most intense signal appears at 2067 cm^−1^ for the 0.3% Pt–H_2_ and 0.1% Pt–H_2_ samples, whereas it shifts to 2074 cm^−1^ for the 0.5% Pt–H_2_ sample. This shift aligns with previous observation of an increase in the frequency of the linearly adsorbed CO with particle size,^53^ consistent with stronger dipole–dipole coupling of CO molecules on larger particles. Secondly, the shoulder associated with terrace sites at 2090 cm^−1^ is clearly observed only for the 0.3% Pt–H_2_ sample.

To explain these unexpected observations, CO adsorption experiments with a small addition of CO dose are conducted to detect any signals that may be hidden by the main peaks due to high CO coverage (Fig. S9). These experiments reveal the presence of signals corresponding to terrace sites at 2095 cm^−1^ also in the spectrum of the 0.5% Pt–H_2_ sample and highlight a growing large band (∼1845 cm^−1^) under the numerous peaks for the IWI–H_2_ sample underlining the presence of bridged CO. Comparing the reduced 0.1% Pt–H_2_ and IWI–H_2_ samples, the shape of the peaks is different, with a wider band for IWI–H_2_ centred around 2050 cm^−1^. Since a larger proportion of clusters is observed on this material, this broader signal is likely due to CO linearly adsorbed on steps of Pt clusters.^63^

One may anticipate consequences for the catalytic properties of these systems. The shift in CO frequency observed for these SACs may indicate a distinct behavior in the catalytic oxidation of CO to CO_2_. While on conventional materials, clusters are active for the catalytic oxidation of CO to CO_2_ and single atoms are not,^35^ the infrared CO signal shifts measured herein for the SOMC materials suggest alternative adsorption sites that may exhibit unique catalytic properties.

## Conclusion

Using surface organometallic chemistry with the MeCpPtMe_3_ organometallic precursor, the preparation of highly dispersed platinum catalysts supported on a needle-like γ-Al_2_O_3_ is achieved, which exhibits different particle size distributions and smaller subnanometric cluster sizes with respect to a sample obtained by the conventional incipient wetness impregnation synthesis method. MeCpPtMe_3_ is shown to be strongly adsorbed but not grafted by protolysis after the deposition procedure employed. Only in the course of further calcination, methane is detected, which may be formed by protolysis with surface hydroxyl groups or other mechanisms. In light of *in situ* infrared experiments, decomposition pathways of the ligands are proposed to explain the formation of platinum catalysts under oxidative and reductive atmospheres. Methane and CO_2_ are detected along calcination, but not under reduction conditions. In the latter case, observations from FTIR are compatible with the formation of Pt clusters.

Notably, a material presenting mainly platinum single atoms on its surface is obtained at 0.1% platinum loading using SOMC. This sharply contrasts with what is observed in a reference sample prepared by incipient wetness impregnation of Pt(NH_3_)_4_(NO_3_)_2_, revealing a larger proportion of Pt single atoms when SOMC is used. Noteworthy, both oxidative atmosphere and low platinum densities are needed to prepare single Pt atoms on alumina. Characterization of reduced materials by microscopy and CO adsorption shows similar particle size, lower than 0.8 nm, regardless of platinum density, when materials are prepared by SOMC. While the size distribution obtained after calcination is strongly dependent on the loading, Pt density appears to have minimal influence on particle size under reductive conditions.

Overall, the use of controlled conditions, including at the deposition step, here using SOMC (organometallic precursor in the absence of water), favours the formation of single atoms or smaller particles (clusters) on alumina. This highlights new perspectives in tuning the catalytic properties of Pt/γ-Al_2_O_3_ catalysts from the proper selection of synthesis methods.

## Author contributions

MC: investigation, formal analysis, writing – original draft, writing – review & editing; MR and VR: formal analysis, supervision, writing – review & editing; IC, ALT, and JP: investigation; AC, CB, ChC, and CCh: conceptualization, formal analysis, supervision, writing – review & editing.

## Conflicts of interest

There are no conflicts to declare.

## Supplementary Material

SC-016-D5SC04893A-s001

## Data Availability

The data supporting this article have been included as part of the supplementary information (SI). Supplementary information: experimental section, characterization of needle alumina by TEM, XRD and BET, additional FTIR and microscopy data. See DOI: https://doi.org/10.1039/d5sc04893a.

## References

[cit1] Russell A., Epling W. S. (2011). Catal. Rev..

[cit2] Sattler J. J. H. B., Ruiz-Martinez J., Santillan-Jimenez E., Weckhuysen B. M. (2014). Chem. Rev..

[cit3] Pham Minh D., Oudart Y., Baubet B., Verdon C., Thomazeau C. (2009). Oil & Gas Science and Technology–Rev. IFP.

[cit4] Le Goff P.-Y. , Kostka W. and Ross J., Catalytic Reforming, 2017

[cit5] Avenier P., Bazer-Bachi D., Bazer-Bachi F., Chizallet C., Deleau F., Diehl F., Gornay J., Lemaire É., Moizan-Basle V., Plais C., Raybaud P., Richard F., Lacombe S. (2016). Oil & Gas Science and Technology–Rev. IFP.

[cit6] Runnebaum R. C., Lobo-Lapidus R. J., Nimmanwudipong T., Block D. E., Gates B. C. (2011). Energy Fuels.

[cit7] Besson M., Gallezot P., Pinel C. (2014). Chem. Rev..

[cit8] Chen S., Wojcieszak R., Dumeignil F., Marceau E., Royer S. (2018). Chem. Rev..

[cit9] Auer F., Blaumeiser D., Bauer T., Bösmann A., Szesni N., Libuda J., Wasserscheid P. (2019). Catal. Sci. Technol..

[cit10] D'Ambra F., Gébel G. (2023). Sci. Tech. Energ. Transition.

[cit11] Sun J., Dong J., Gao L., Zhao Y.-Q., Moon H., Scott S. L. (2024). Chem. Rev..

[cit12] Zhang F., Zeng M., Yappert R. D., Sun J., Lee Y.-H., LaPointe A. M., Peters B., Abu-Omar M. M., Scott S. L. (2020). Science.

[cit13] Marceau E. , Carrier X., Che M., Clause O., Marcilly C., Handbook of Heterogeneous Catalysis: Ion Exchange and Impregnation, 2008

[cit14] Spieker W. A., Regalbuto J. R. (2001). Chem. Eng. Sci..

[cit15] Hutchings G. J. and Védrine J. C., Heterogeneous Catalyst Preparation, Basic Principles in Applied Catalysis, ed. M. Baerns, Springer, Berlin, Heidelberg, 2004, vol. 75

[cit16] de Jong K. P. , Synthesis of Solid Catalysts: Deposition Precipitation, Wiley, Weinheim, 2009

[cit17] Miller J. (2004). J. Catal..

[cit18] Regalbuto J. R. , Synthesis of solid catalysts: Electrostatic Adsorption, Wiley, Weinheim, 2009

[cit19] Héroguel F., Gebert D., Detwiler M. D., Zemlyanov D. Y., Baudouin D., Copéret C. (2014). J. Catal..

[cit20] Womes M., Cholley T., Le Peltier F., Morin S., Didillon B., Szydlowski-Schildknecht N. (2005). Appl. Catal. A Gen..

[cit21] Liu S., Tan J. M., Gulec A., Crosby L. A., Drake T. L., Schweitzer N. M., Delferro M., Marks L. D., Marks T. J., Stair P. C. (2017). Organometallics.

[cit22] McCullough K. E., Peczak I. L., Kennedy R. M., Wang Y.-Y., Lin J., Wu X., Paterson A. L., Perras F. A., Hall J., Kropf A. J., Hackler R. A., Shin Y., Niklas J., Poluektov O. G., Wen J., Huang W., Sadow A. D., Poeppelmeier K. R., Delferro M., Ferrandon M. S. (2023). J. Mater. Chem. A.

[cit23] Camacho-Bunquin J., Ferrandon M., Sohn H., Yang D., Liu C., Ignacio-de Leon P. A., Perras F. A., Pruski M., Stair P. C., Delferro M. (2018). J. Am. Chem. Soc..

[cit24] Copéret C. (2019). Acc. Chem. Res..

[cit25] Gould T. D., Lubers A. M., Corpuz A. R., Weimer A. W., Falconer J. L., Medlin J. W. (2015). ACS Catal..

[cit26] Dasgupta N. P., Liu C., Andrews S., Prinz F. B., Yang P. (2013). J. Am. Chem. Soc..

[cit27] Lu J., Low K.-B., Lei Y., Libera J. A., Nicholls A., Stair P. C., Elam J. W. (2014). Nat. Commun..

[cit28] van Daele M., Detavernier C., Dendooven J. (2018). Phys. Chem. Chem. Phys..

[cit29] Vandalon V., Mackus A. J. M., Kessels W. M. M. (2022). J. Phys. Chem. C.

[cit30] Aksoylu A. E., Faria J. L., Pereira M., Figueiredo J. L., Serp P., Hierso J.-C., Feurer R., Kihn Y., Kalck P. (2003). Appl. Catal. A Gen..

[cit31] Thurier C., Doppelt P. (2008). Coord. Chem. Rev..

[cit32] Copéret C., Comas-Vives A., Conley M. P., Estes D. P., Fedorov A., Mougel V., Nagae H., Núñez-Zarur F., Zhizhko P. A. (2016). Chem. Rev..

[cit33] Docherty S. R., Rochlitz L., Payard P.-A., Copéret C. (2021). Chem. Soc. Rev..

[cit34] Oviroh P. O., Akbarzadeh R., Pan D., Coetzee R. A. M., Jen T.-C. (2019). Sci. Technol. Adv. Mater..

[cit35] Dessal C., Len T., Morfin F., Rousset J.-L., Aouine M., Afanasiev P., Piccolo L. (2019). ACS Catal..

[cit36] Kwak J. H., Hu J., Mei D., Yi C.-W., Kim D. H., Peden C. H. F., Allard L. F., Szanyi J. (2009). Science.

[cit37] Batista A. T. F., Baaziz W., Taleb A.-L., Chaniot J., Moreaud M., Legens C., Aguilar-Tapia A., Proux O., Hazemann J.-L., Diehl F., Chizallet C., Gay A.-S., Ersen O., Raybaud P. (2020). ACS Catal..

[cit38] Bradley S. A., Sinkler W., Blom D. A., Bigelow W., Voyles P. M., Allard L. F. (2012). Catal. Lett..

[cit39] Copéret C., Comas-Vives A., Conley M. P., Estes D. P., Fedorov A., Mougel V., Nagae H., Núñez-Zarur F., Zhizhko P. A. (2016). Chem. Rev..

[cit40] Digne M., Sautet P., Raybaud P., Euzen P., Hervé T. (2004). J. Catal..

[cit41] Völker L. A., Meyet J., Berkson Z. J., Rochlitz L., van Bokhoven J. A., Copéret C. (2022). J. Phys. Chem. C.

[cit42] Krokidis X., Raybaud P., Gobichon A.-E., Rebours B., Euzen P., Toulhoat H. (2001). J. Phys. Chem. B.

[cit43] Ali S., Abbas Y., Zuhra Z., Butler I. S. (2019). Nanoscale Adv..

[cit44] Bekele E. A., Korsa H. A., Desalegn Y. M. (2024). Sci. Rep..

[cit45] Li D.-Y., Lin Y.-S., Li Y.-C., Shieh D.-L., Lin J.-L. (2008). Microporous Mesoporous Mater..

[cit46] Zhou S., Antonietti M., Niederberger M. (2007). Small.

[cit47] Kessels W. M. M., Knoops H. C. M., Dielissen S. A. F., Mackus A. J. M., van de Sanden M. C. M. (2009). Appl. Phys. Lett..

[cit48] Gallinella E., Fortunato B., Mirone P. (1967). J. Mol. Spectrosc..

[cit49] Morfin F., Dessal C., Sangnier A., Chizallet C., Piccolo L. (2024). ACS Catal..

[cit50] Couble J., Bianchi D. (2017). J. Catal..

[cit51] Busca G., Lamotte J., Lavalley J. C., Lorenzelli V. (1987). J. Am. Chem. Soc..

[cit52] Erkens I. J. M., Mackus A. J. M., Knoops H. C. M., Smits P., van de Ven T. H. M., Roozeboom F., Kessels W. M. M. (2012). ECS J. Solid State Sci. Technol..

[cit53] Sangnier A., Genty E., Iachella M., Sautet P., Raybaud P., Matrat M., Dujardin C., Chizallet C. (2021). ACS Catal..

[cit54] Candy J.-P., Fouilloux P., Primet M. (1978). Surf. Sci..

[cit55] Carosso M., Vottero E., Lazzarini A., Morandi S., Manzoli M., Lomachenko K. A., Ruiz M. J., Pellegrini R., Lamberti C., Piovano A., Groppo E. (2019). ACS Catal..

[cit56] Paleček D., Tek G., Lan J., Iannuzzi M., Hamm P. (2018). J. Phys. Chem. Lett..

[cit57] Comas-Vives A., Valla M., Copéret C., Sautet P. (2015). ACS Cent. Sci..

[cit58] James E., Browning N. (1999). Ultramicroscopy.

[cit59] Nellist P. D., Pennycook S. J. (1996). Science.

[cit60] Zhang Z., Zhu Y., Asakura H., Zhang B., Zhang J., Zhou M., Han Y., Tanaka T., Wang A., Zhang T., Yan N. (2017). Nat. Commun..

[cit61] Wang H., Dong J., Allard L. F., Lee S., Oh S., Wang J., Li W., Shen M., Yang M. (2019). Appl. Catal. B Environ..

[cit62] Murata K., Kurimoto N., Yamamoto Y., Oda A., Ohyama J., Satsuma A. (2021). ACS Appl. Nano Mater..

[cit63] Haaland D. M. (1987). Surf. Sci..

