# Supplementary material for: Tuning cluster size down to single atoms on Pt/γ-Al2O3 catalysts via surface organometallic chemistry
Source: Chem Sci. 2025 Oct 23;16(47):22748–56. doi: 10.1039/d5sc04893a (PMC12577013; doi:10.1039/d5sc04893a)
Supplement: SC-016-D5SC04893A-s001 [file SC-016-D5SC04893A-s001.pdf]

Supplementary Information (SI) for Chemical Science.
This journal is © The Royal Society of Chemistry 2025

Supporting Information for:

Tuning cluster size down to single atom on Pt/γ-Al_2O_3 catalysts via surface organometallic chemistry

Martin Cotoni,[1] Mickael Rivallan,[1,] Isabelle Clemençon,[1] Virgile Rouchon,[2] Anne-Lise Taleb,[1] Julie Poulizac,[1] Amandine Cabiac,[1] Christophe Bouchy,[1] Christophe Copéret,[2,*] Céline Chizallet[1,*]*

[1] IFP Energies nouvelles, Rond-point de l'échangeur de Solaize, BP3, 69360 Solaize, France

[2] Department of Chemistry and Applied Biosciences, ETH Zürich, Zürich CH-8093, Switzerland

Corresponding authors:

mickael.rivallan@ifpen.fr, ccoperet@inorg.chem.ethz.ch, celine.chizallet@ifpen.fr

Figure S1. Characterization of needles: diffractogram of boehmite (a) and of calcined alumina, (b) TEM of needle-shaped alumina (c-d), (e) N_2 adsorption isotherm of needles shaped alumina. 5
Figure S2. Proton NMR of the grafting test of the organometallic precursor after a night of stirring 6
Figure S3. Comparison of FTIR spectra of 0.1%Pt before and after air exposure with focus on (a) surface hydroxyls vibration area, (b) 1000-2000 cm^{-1} range, (c) CO vibration area. 7
Figure S4. Deconvolution of the FTIR spectrum of the 0.1%Pt sample at room temperature after exposition to air, in the 1100-1350 cm^{-1} range. 7
Figure S5. Infrared spectra with a focus on the range 2200-1000 cm^{-1} of the 0.1%Pt sample during (a) calcination and (b) reduction 8
Figure S6. Histograms of mean diameter observed on (a)-(b) 0.3-0.5%Pt-Air, and (c) IWI-H_2 and (d)-(e)-(f) 0.5-0.3-0.1%Pt-H_2. 10
Figure S7. Dosed CO adsorption monitored by FTIR on (a) 0.5 %Pt-H_2, (b) 0.3 %Pt-H_2, (c) 0.1 %Pt-H_2, (d) IWI-H_2...11

Experimental section

Alumina Preparation

The synthesis of γ-Al_2O_3 needles was adapted from the procedure reported in ref.[1] 350 mL of Milli-Q water was added to a mixture of aluminum acetate (Prolabo) (1 mol/L) and dodecanoic acid (Sigma-Aldrich) (0.5 mol/L). The solution was heated to 180°C for 72 h in a hydrothermal reactor. Resulting boehmite nanocrystals were washed by centrifugation with isopropanol and dried under vacuum (1.10^{-3} bar) before calcination for six hours at 600°C in a muffle furnace with a heating rate of 1 °C/min. The final solid, characterized by X-ray diffraction (XRD), was identified as γ-Al_2O_3 with a specific surface area of 104 m^2/g. A second calcination for eight hours at 500°C with a heating rate of 5°C/min followed by a dehydration under high vacuum (1.10^{-4} mbar) overnight at the same temperature was performed. The dehydrated γ-Al_2O_3 sample was stored in a glove box for further uses. Characterization of the support is available in Figure S1.

Catalyst Preparation

Catalysts were prepared in glove box under an argon atmosphere using conventional Schlenk techniques. Solutions of $MeCpPtMe_3$ (Sigma-Aldrich) were prepared by dissolving the targeted amount of precursor in pentane (details are given in Table S1). These solutions were added drop wise on dehydrated alumina needles suspended in a minimum amount of pentane. The reaction medium was stirred overnight at room temperature. At the end of stirring white alumina turn into a yellow solid in suspension in the solvent. After three washing steps with pentane, the resulting solid was dried under vacuum (1.10^{-1} mbar) at room temperature giving a yellow powder. Three catalysts were prepared according to the described procedure with various target platinum surface densities: 0.03-0.09-0.15 Pt/nm^2 corresponding respectively to Pt loading of 0.1-0.3-0.5wt%. 200 mg of the prepared materials were thermally treated (2°C/min) under dry air (water content < 1 ppm) or hydrogen (Air Product, purity > 99.9992%) flow (2 L/h for both gaseous flows) at the temperature chosen after in situ characterization (see below). Materials will be denoted in the following text as X% Pt (X corresponding to Pt loading). "Air" or "H_2" suffixes will be added to indicate the thermal treatment applied (as an example a material with a loading in weight in Pt of 0.1% which was calcined will be denoted as 0.1% Pt-Air while the reduced one will be denoted as 0.1% Pt-H_2). Concerning the solid prepared with $Pt(NH_3)_4(NO_3)_2$, it was prepared by impregnating 6.1 mg of precursor dissolved in 3.9 ml of distilled water on 3 g of pre-calcined (600 °C) Needles γ-Al_2O_3. The wet solid was dried at 60 °C for 5h in muffle furnace. Dried material was calcined under an air flow (2 L/h) at 450 °C (2 °C/min) for 2h. Material will be denoted as IWI-Air or IWI-H_2 depending on the treatment applied. The Pt real loading of all sample after preparation were titrated by powder X-ray fluorescence on a ^{ARL}Perfom'X (Thermo Scientific) for the 0.5% Pt and 0.3% Pt giving a loading in platinum of 0.54 and 0.29 wt% and on ^{ARL}Quant'X EDXRF Analyser for the 0.1% Pt and IWI samples giving respectively a loading of 0.1 and 0.11 wt % in platinum.

Catalyst characterization

X-ray diffraction (XRD)

Diffractograms were acquired on a Bruker D4 ENDEAVOR device with a Cu Kα source (λ=1.54184 Å) to characterize the synthesized boehmite and alumina. The diffractogram of the boehmite was indexed using the CIF-boehmite file developed by Guzmán-Castillo et al.[2] (space

group *Cmcm*). The diffractogram of alumina was indexed using the CIF-alumina file from Smrock et al.[3] (space group *Fd-3m*). Both diffractograms are represented Figure S1.

Brunauer–Emmett–Teller (BET) surface measurement

BET surfaces were measured by nitrogen physisorption. Isotherm curves were acquired after drying the aluminas at 350°C under vacuum before a pressure build-up under N_2, using a Micrometrics ASAP 2020.

Electron microscopy

Microscopy analysis was carried out using a JEOL JEM-F200 microscope operated in two modes. A parallel beam imaging mode (TEM) was used to characterize the bare alumina support, while High-angle annular dark-field scanning transmission (HAADF-STEM) was performed to characterize alumina-supported Pt after thermal treatments. Both modes were operated at 200 kV. Probe Cs-corrected High-resolution HAADF-STEM analysis was moreover carried out on a JEM-ARM200F 200 kV microscope. This microscope was principally used to characterize the 0.1Pt%-Air and IWI-Air samples. The samples were ground in an agate mortar and deposited without solvent on a 300 mesh holey-carbon-coated copper grid. Pictures were processed using Gatan's DigitalMicrograph software. Particle size histograms and average size were obtained from 200 objects measurement (Figure S6), excepted on samples 0.1%Pt-Air and IWI-Air on the JEM-F200 because of smaller particle sizes and numbers as well as resolution limitations (Figure S7).

Grafting test investigated by ^{1}H Nuclear Magnetic Resonance (NMR)

In a glove box, a dried sealed NMR tube was filled with 20 mg of dehydrated alumina and 5 mg of sublimed $FeCp_2$ used as an internal standard (98 %, Thermo Fisher Scientific), were suspended in C_6D_6. Then the mixture was cooled down in freezer until the solidification of the mixture. After cooling, 4 mg of $MeCpPtMe_3$ was dissolved in a few amounts of C_6D_6 and added to the NMR tube while the suspension of alumina and of $FeCp_2$ remained frozen. Eventually, the NMR tube was filled completely with C_6D_6 before a night of stirring. The mixture was then analyzed by proton NMR spectroscopy with a sequence of 16 scans on a 300 MHz Bruker Ultra shield spectrometer.

Infra-red spectroscopy

FTIR spectra were recorded in transmission mode with an IS50 spectrometer (Thermo-Optek) in the range 400 cm^{-1} to 4000 cm^{-1} with 64 scans. For FTIR experiments in static conditions, self-supported pellets (weighted around 20 mg) were prepared in a glove box under inert atmosphere and analyzed in an infrared cell. CO adsorption was monitored on ex situ treated catalysts in the same conditions. The cell was evacuated under high vacuum (1.10^{-5} mbar) and filled with CO to reach complete saturation of the surface. Spectra were then acquired after a complete evacuation of CO atmosphere using high vacuum.

In situ analyses were carried out using a FTIR device developed by Visio technics. The device does not allow to load the supported pellets without exposing the sample to air. FTIR analysis of the evolution of the surface of a self-supported pellet weighted around 8 mg was performed, exposed to a gaseous flow (2 L/h) at atmospheric pressure, as a function of temperature and time. A ramp in temperature was settled at 2 °C/min from room temperature to 450 °C with a step of 2 h at 450 °C. Spectra were obtained in the same way as conventional FTIR.

Characterization of the support

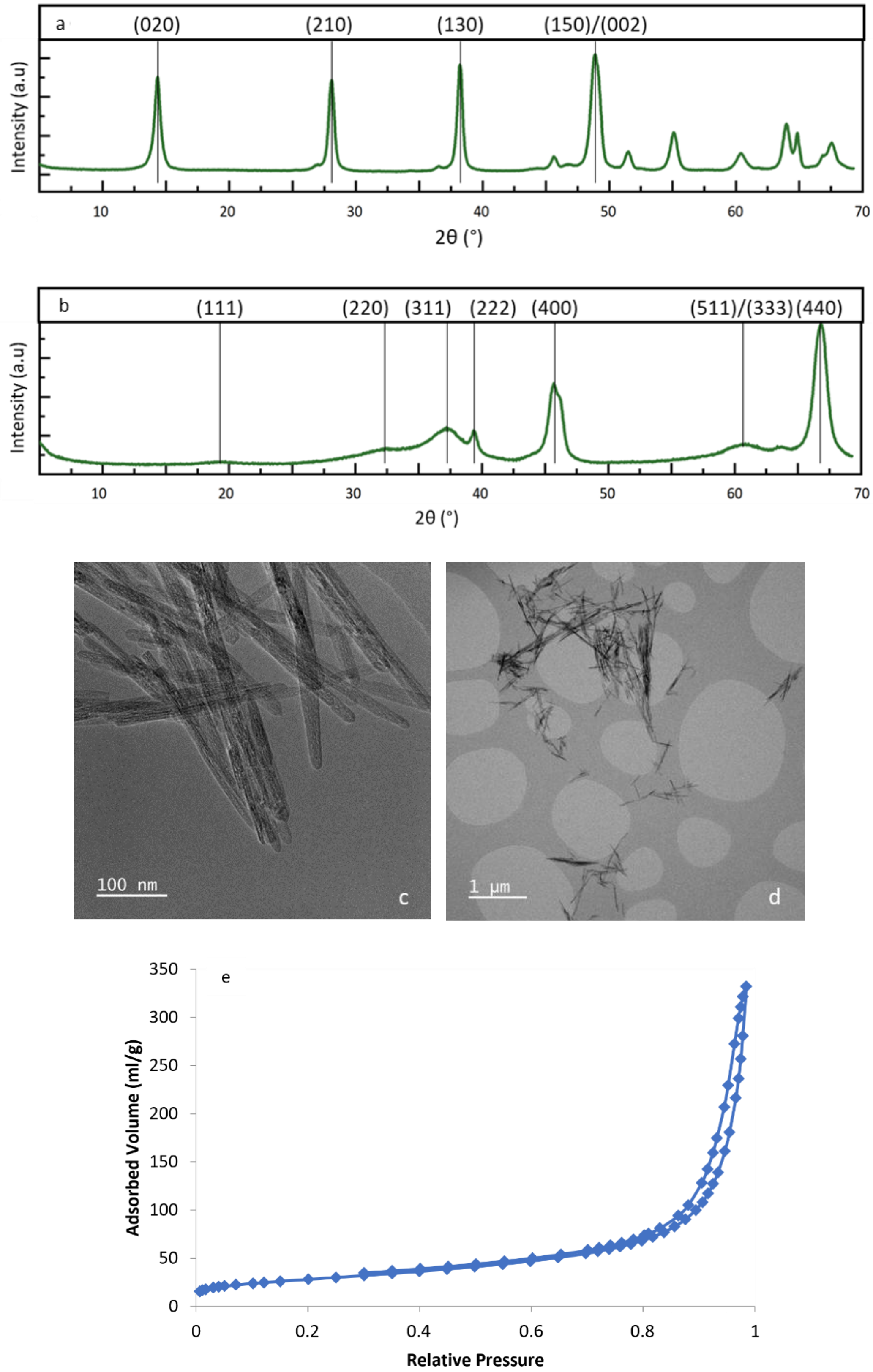

Figure S1. Characterization of needles: diffractogram of boehmite (a) and of calcined alumina, (b) TEM of needle-shaped alumina (c-d), (e) N_2 adsorption isotherm of needles shaped alumina.

Grafting

Table S1. Summary of amount of precursor and alumina used for the preparation of the materials

Pt loading (%wt)	Mass of Precursor (mg) / volume of pentane (mL)	Mass of alumina (mg)
0.5	5.5 / 5	663
0.3	4.0 / 5	800
0.1	3.2 / 5	1890

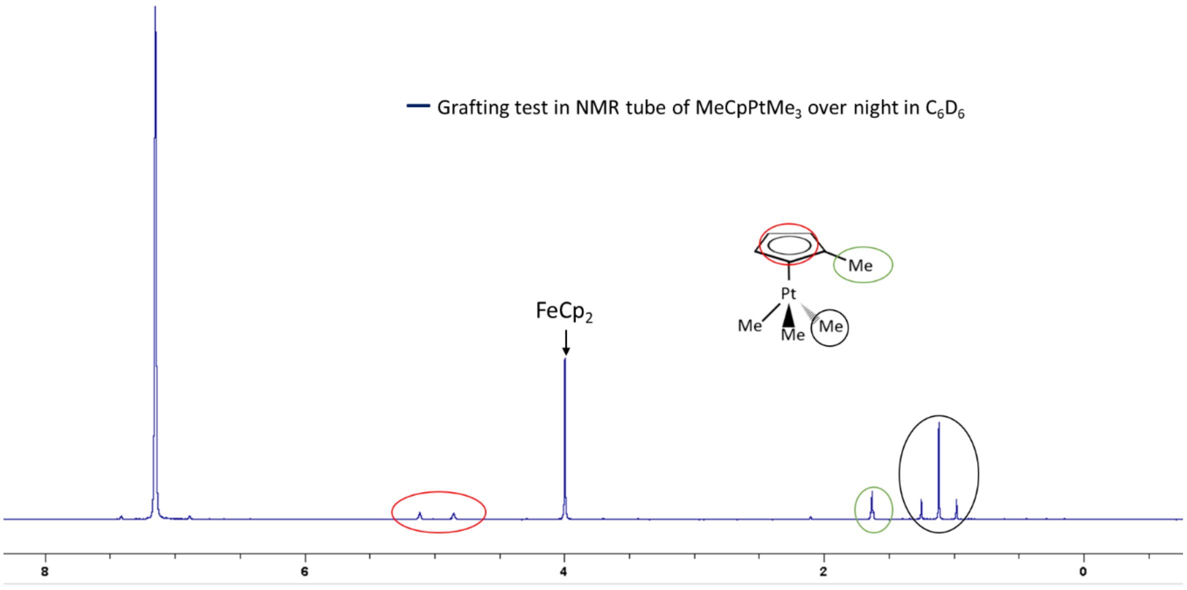

Figure S2. Proton NMR of the grafting test of the organometallic precursor after a night of stirring

Complementary FTIR data

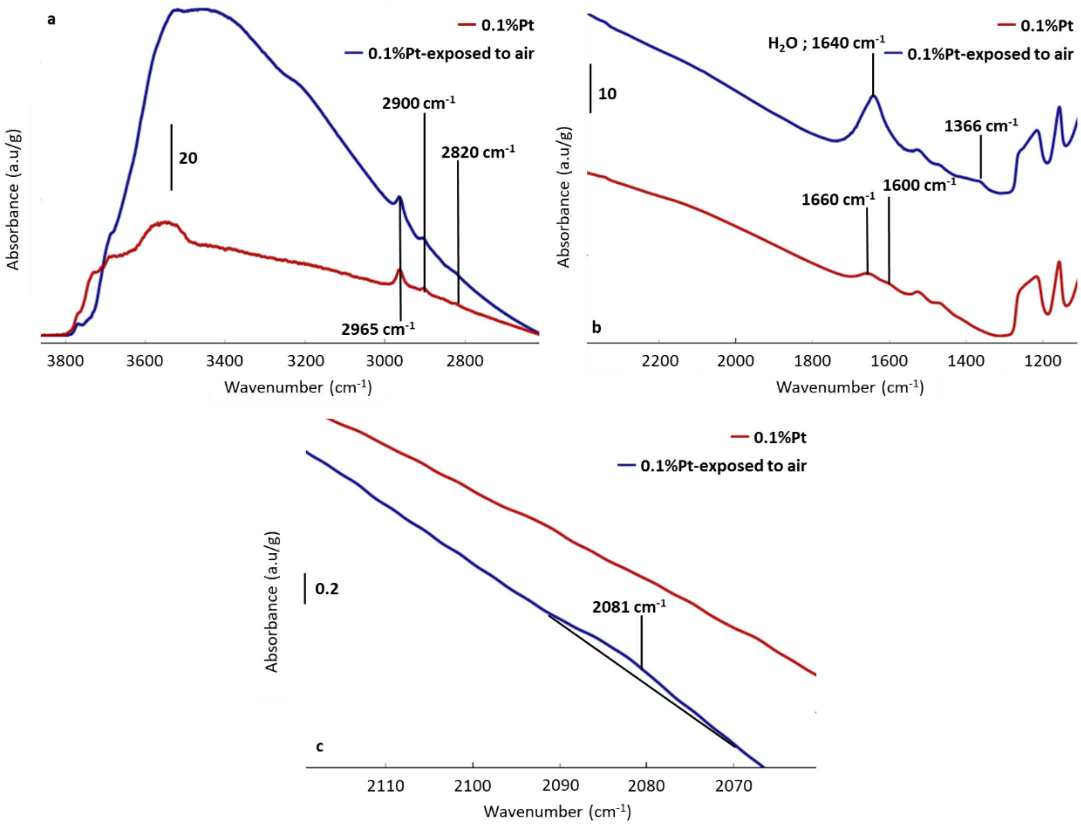

Figure S3. Comparison of FTIR spectra of 0.1%Pt before and after air exposure with focus on (a) surface hydroxyls vibration area, (b) 1000-2000 cm^{-1} range, (c) CO vibration area.

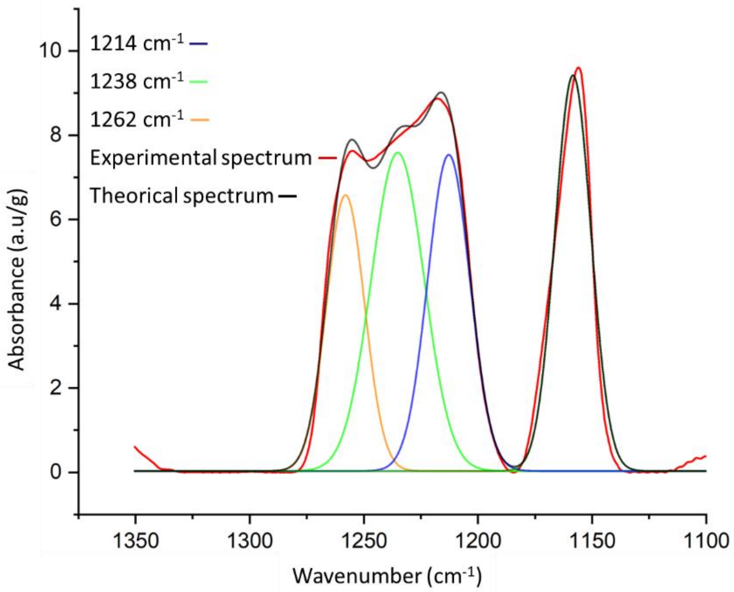

Figure S4. Deconvolution of the FTIR spectrum of the 0.1%Pt sample at room temperature after exposition to air, in the 1100-1350 cm^{-1} range.

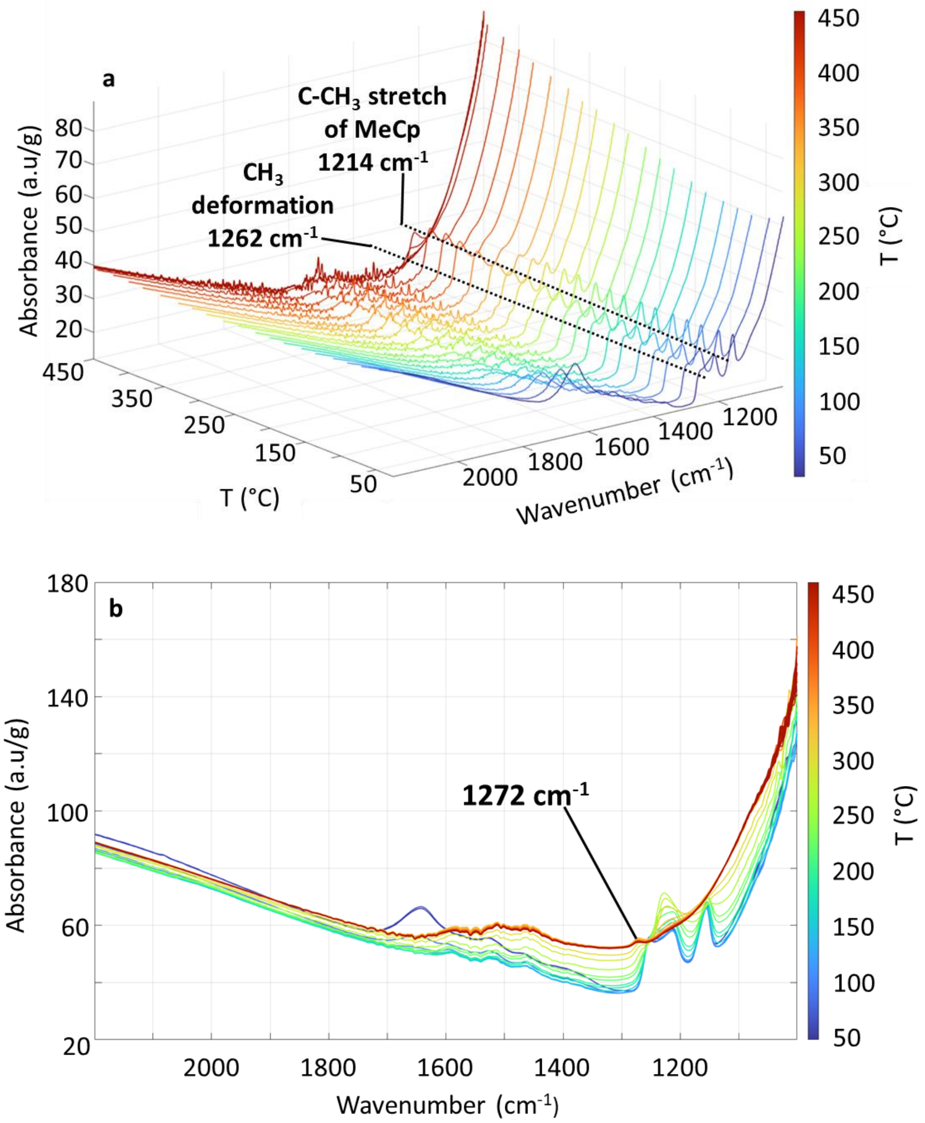

Figure S5. Infrared spectra with a focus on the range 2200-1000 cm^{-1} of the 0.1%Pt sample during (a) calcination and (b) reduction

Under calcination conditions, between room temperature and 120 °C, the desorption of water and a decrease of the weak signals attributed to CO occur. At 150°C, CO_2 appears (ν_{as}(COO); 2403-2247 cm^{-1}), while the decrease of the integrated areas between 2988-2865 cm^{-1} (ν(C-H)) and 1274-1186 cm^{-1} (CH_3 deformation and C-CH_3 stretch) is substantial from 170°C. Chemisorbed methane (ν(C-H) at 3016 cm^{-1} [4,5]) appears from 200 °C, concomitantly with a decrease of ν(C-H), CH_3 deformation/C-CH_3 stretch bands. ν(C-H) modes of alkyls vanish at 310 °C. The bands corresponding to CH_3 wagging decrease from 250°C and vanish at 360 °C, where methane production reaches a plateau, same as CH_3 deformation/C-CH_3 stretch bands. Finally, the later bands (1274-1186 cm^{-1}) undergo between 380 and 450 °C a sharper decrease, reaching zero intensity at the end of the plateau at 450 °C while the signal of methane starts to decrease during the plateau. This two-step decrease of the signal integrated between 1274-1186 cm^{-1} is due to the overlapping of the signals assigned to CH_3 deformation and C-CH_3 stretch, with first a decrease of CH_3 deformation between 170 °C and 330 °C and then a decrease of the C-CH_3 stretch during the end of the treatment, as illustrated on Figure S5-a. Throughout all the described steps, the signal of CO_2 continues to rise.

HR-STEM

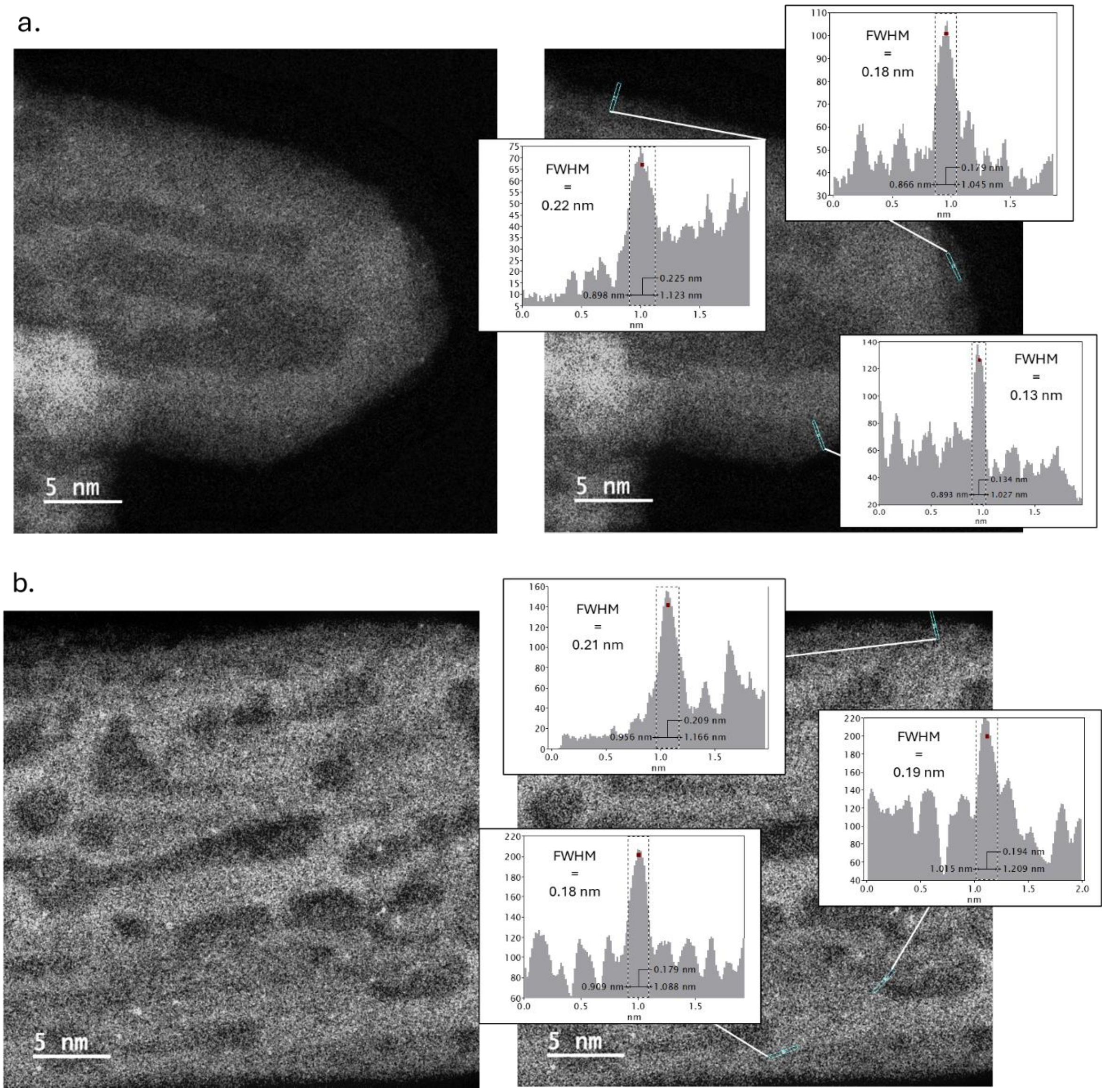

Figure S6. HAADF-STEM images obtained with the JEM-F200 microscope of (a) 0.1%Pt-Air, and (b) IWI-Air samples. On the right, STEM-HAADF intensity profiles are given are given along specific directions where objects are imaged. The full width at half maximum is in the selected cases clearly lower than 0.25 nm, making it possible to conclude that these objects are single atoms.

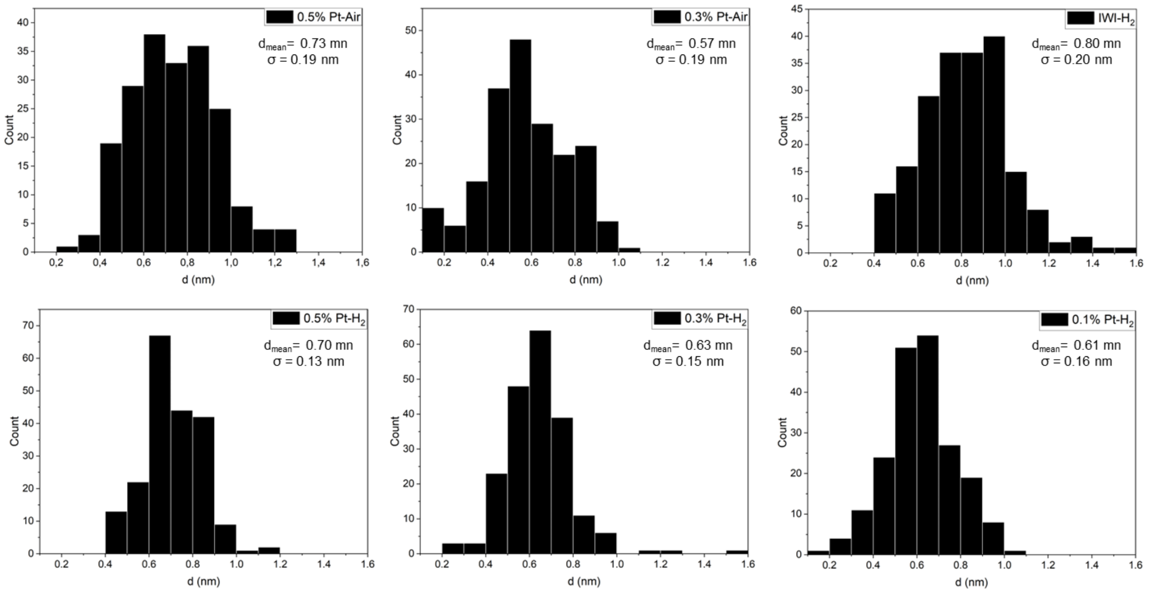

Figure S7. Histograms (n=200) of particle size with their standard deviations observed on the sample observed with the JEM-F200 microscope (except IWI-Air and 0.1%Pt-Air, see Figure S7). Mean diameter and standard deviation are obtained from the data fitting with a normal distribution function.

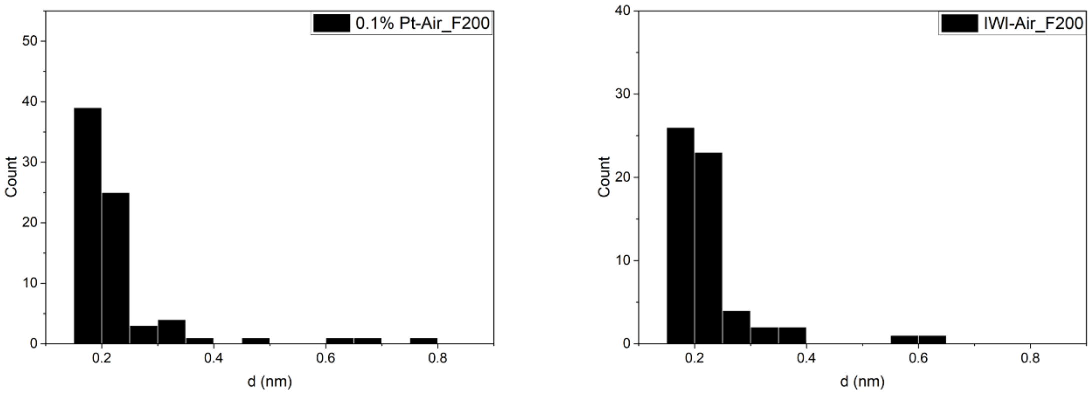

Figure S8. Histograms (n=60 for IWI-Air and n=76 for 0.1%Pt-Air) of particle size observed with the JEM-F200 microscope. No distribution function fitting was performed due to the limited number of objects.

CO adsorption

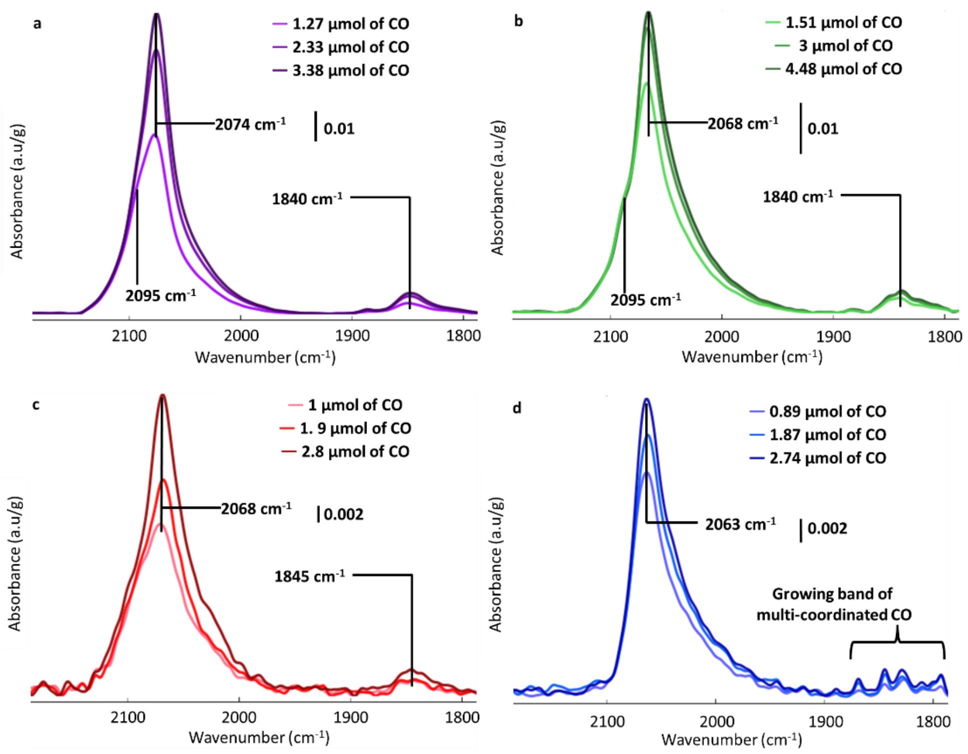

Figure S9. Dosed CO adsorption monitored by FTIR on (a) 0.5% Pt-H_2, (b) 0.3% Pt-H_2, (c) 0.1% Pt-H_2, (d) IWI-H_2

References

1. L. A. Völker, J. Meyet, Z. J. Berkson, L. Rochlitz, J. A. van Bokhoven and C. Copéret, *J. Phys. Chem. C*, 2022, **126**, 6351.
2. M. L. Guzmán-Castillo, X. Bokhimi, A. Toledo-Antonio, J. Salmones-Blásquez and F. Hernández-Beltrán, *J. Phys. Chem. B*, 2001, **105**, 2099.
3. L. Smrcok, V. Langer and J. Krestan, *Acta crystallographica. Section C, Crystal structure communications*, 2006, **62**, i83-4.
4. G. Busca, J. Lamotte, J. C. Lavalley and V. Lorenzelli, *J. Am. Chem. Soc.*, 1987, **109**, 5197.
5. J. Joubert, A. Salameh, V. Krakoviack, F. Delbecq, P. Sautet, C. Copéret and J. M. Basset, *J. Phys. Chem. B*, 2006, **110**, 23944.
